# Coping with compliance during take-off and landing in the diamond dove (*Geopelia cuneata*)

**DOI:** 10.1371/journal.pone.0199662

**Published:** 2018-07-25

**Authors:** Kristen E. Crandell, Austin F. Smith, Ondi L. Crino, Bret W. Tobalske

**Affiliations:** 1 Field Research Station at Fort Missoula, Division of Biological Sciences, University of Montana, Missoula, MT, United States of America; 2 School of Biological Sciences, Bangor University, Bangor, Gwynedd, United Kingdom; 3 Centre for Integrative Ecology, Deakin University, Victoria, Australia; Brown University, UNITED STATES

## Abstract

The natural world is filled with substrates of varying properties that challenge locomotor abilities. Birds appear to transition smoothly from aerial to terrestrial environments during take-offs and landings using substrates that are incredibly variable. It may be challenging to control movement on and off compliant (flexible) substrates such as twigs, yet birds routinely accomplish such tasks. Previous research suggests that birds do not use their legs to harness elastic recoil from perches. Given avian mastery of take-off and landing, we hypothesized that birds instead modulate wing, body and tail movements to effectively use compliant perches. We measured take-off and landing performance of diamond doves (*Geopelia cuneata (N = 5*) in the laboratory and perch selection in this species in the field (N = 25). Contrary to our hypothesis, doves do not control take-off and landing on compliant perches as effectively as they do on stiff perches. They do not recover elastic energy from the perch, and take-off velocities are thus negatively impacted. Landing velocities remain unchanged, which suggests they may not anticipate the need to compensate for compliance. Legs and wings function as independent units: legs produce lower initial velocities when taking off from a compliant substrate, which negatively impacts later flight velocities. During landing, significant stability problems arise with compliance that are ameliorated by the wings and tail. Collectively, we suggest that the diamond dove maintains a generalized take-off and landing behavior regardless of perch compliance, leading us to conclude that perch compliance represents a challenge for flying birds. Free-living diamond doves avoid the negative impacts of compliance by preferentially selecting perches of larger diameter, which tend to be stiffer.

## Introduction

Natural habitats are full of structural variation that imposes constraints upon locomotor performance. Vegetation varies in orientation, roughness, size, and compliance (i.e., flexibility, or the opposite of stiffness; compliant substrates easily deform as a function of applied force). For example, the compliance of branches varies 4-fold within a genus of trees in a single forest [1]. Natural perch variation can directly impact locomotor capacity. As an example, a slight change in the angle of a substrate (from 0 to +10 degrees) causes a 46% decrease in running velocity in geckos that have their adhesive system deployed [2].

Substrate compliance poses a unique challenge. As an organism moves, work is done on the substrate. If that substrate is compliant, that work is absorbed by the substrate–resulting in lower net work for the animal to effectively launch from the substrate during running and leaping [3]. Some compliant substrates, such as tree branches, are elastic, allowing the effective work to be recaptured during a recoil. For such substrates, shape, diameter, and length play a role in the compliance of the substrate [4]. Given the high degree of variation in substrates, it is reasonable to predict that organisms have adapted a strategy to accommodate structural compliance within habitats.

Response to substrate compliance appears to vary among species. Models of humans, bush-babies, and insects suggest that modifying kinematics on substrates of various compliance can change jump height [5]. Likewise, orangutans are capable of capitalizing on whole-tree compliance by using trees as an “external spring” converting the potential energy of the tree into kinetic energy to move across the canopy [6]. Similarly, arboreal tree frogs recover elastic energy from compliant substrates during take-off as the perch recoils [7]. However, in *Anolis* lizards, compliant perches constrain initial jump velocity [8], and individuals prefer stiffer substrates [9]. Whether flying organisms capitalize on similar modifications in kinematics to benefit from compliant substrates remains unknown.

Reactions to compliance may differ between terrestrial and volant organisms. Shifting between a terrestrial and aerial environment appears seamless for many flying birds, yet actually requires enormous amounts of power [10]. In birds, take-off and landing offer potential instances of selective pressure. In many species, avoiding predators, courting mates, and foraging rely in part on a successful take-off. A precise landing could also aid in avoiding wing or whole-body damage. Thus, a logical prediction is that birds should take-off and land quickly to minimize energy expenditure and avoid damage.

Anatomical and physiological wing and leg “modules” in birds, which operate independently during terrestrial and aerial locomotion, were thought to function at the same time during transition stages [11]. However, recent work has established that during take-off the legs and wings function temporally independently—one after the other. Despite this independence, both contribute to the take-off. The legs contribute most to initial acceleration in take-off, and the wings follow with significant forces only after the feet leave the substrate [12]. During landing, the wings contribute the majority of the deceleration prior to leg interaction with the substrate [13]. Control and stability during these phases is potentially challenged by large variation in compliance of natural perches. Little is known concerning how birds accommodate such a variety of structures during transitions in spite of how this ties together the ecology of these animals with their primary locomotor style.

During take-off birds do not appear to adjust their leg forces to accommodate variation in perch compliance [14]. However, two other adjustments may occur to maximize take-off velocity. The first is that birds may adjust the timing over which the force is applied. As velocity = (force * time) / mass, if force remains constant, the remaining variable that can be adjusted for greater velocity is time. In contrast, volant organisms may compensate for lost performance from the hindlimbs in flight. Birds are known to modulate their force production following take-off by engaging their wings to different degrees [12]. Therefore, even if energy is lost to the perch, causing a lower or negligible initial jump velocity (as suggested in European Starlings *Sturnus vulgaris*; [14]), volant organisms may be able to compensate quickly in the air. Given that hummingbirds modify take-off velocities via both wing and leg contributions depending on motivational state [15], we predict that any initial take-off velocity lost due to compliance [14] will be overcome in the air.

During landing, the leg forces are smaller than those during take-off [12, 13]. In comparison to terrestrial organisms, aerial organisms capitalize on aerodynamic decelerations prior to landing, and so impact forces are lower. As such, volant organisms may be able to prepare prior to landing to accommodate variation in substrate properties. In particular, birds may approach a known perch at higher velocity, expending less energy in decelerating in flight, if instead work is done by the compliant perch, rather than the legs, on landing. Pigeons approach a novel perch at a much lower flight velocity than a well-known perch [16], suggesting that variation in preparation for landing on known substrates occurs in birds and that landings on unknown substrates may be performed sub-optimally in terms of energy output in favor of improved safety.

**Fig 1:**
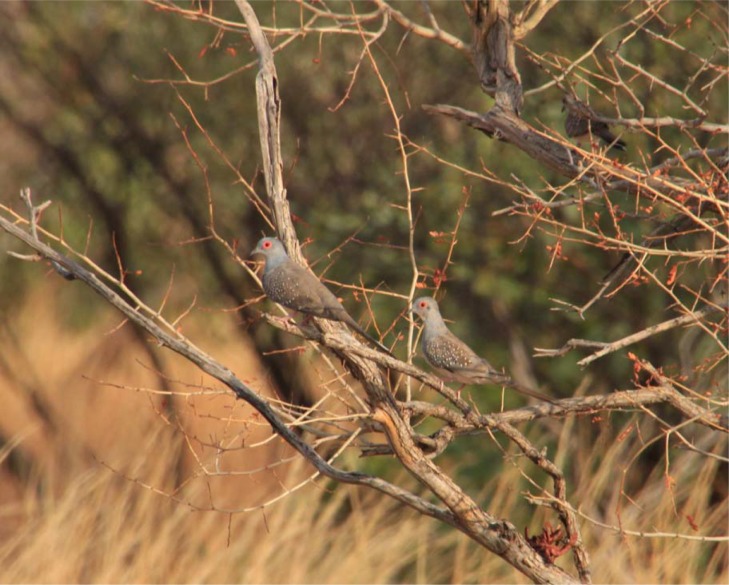
Free-living diamond doves (*Geopelia cuneata*) in Australia. Photo by Ondi Crino.

Given the ecological significance of compliant substrate use and the differences between the forces produced during take-off and landing in terrestrial and aerial organisms, and the seeming mastery of this variation by flying birds, we hypothesized that birds should modulate wing, body and tail kinematics to accomplish similar take-off and landing performance regardless of substrate compliance. To test this, we used diamond doves (Fig 1; *Geopelia cuneata*) in the laboratory and field to ask: (1) How are take-off and landing velocities affected by perch compliance? (2) Do birds coordinate leg and wing modules to accommodate perch compliance? (3) Do free-living birds select perches to avoid or enhance potential effects of compliance?

## Materials & methods

### Perch use

Five diamond doves (*Geopelia cuneata*) were used in this study. This species was selected due to availability from the pet trade and a rich background on the mechanics of take-off and landing in the doves from recent studies [12, 13]. All procedures were approved by the Institutional Animal Care and Use Committee at the University of Montana (043-10BTDBS-101210). Birds were trained to fly horizontally in the laboratory between two perches two meters apart by starting with perches close together and slowly increasing the distance apart with successive flights, as in Provini et. al [12]. During training, birds randomly experienced each of three perch types for a minimum of ten flights before substrates were changed. Training occurred daily for ten days prior to data collection. Take-offs and landings were generally prompted by a wave of a hand behind the animal.

During experiments, perches were mounted to a custom 3-axis force plate (Bertec; Bertec Corp. Columbus, OH, USA). Forces were amplified 10x (Bertec AM6800) and recorded at 500 Hz using Chart software (v4.5, AD Instruments, Inc., Colorado Springs, CO, USA).

We varied perch compliance (the opposite of flexural stiffness, *EI*) using wood (*EI* = 0.39 Nm^2^), aluminum (*EI* = 2.12 Nm^2^), or steel (*EI* = 6.14 Nm^2^) perches, each 5 mm in diameter. Flexural stiffness values were calculated given the shape and material properties of the beam. Estimated deflection for these perches, with a 50 g bird perched at the take-off location, 40 cm from the point of attachment, were 2.6 cm, 0.5 cm, and 0.2 cm, respectively. Perches were kept in their natural coloration, and were distinguishable visually.

Prior to experimental trials, birds were given five to ten practice transitions on each substrate, in order to re-familiarize them with the substrate. No evidence for learning (i.e., a consistent change in body velocity or behavior throughout experiments) was apparent in the data. Ten trials were collected per substrate, per bird, for both take-off and landing (60 trials total per bird). The order substrates were used was randomized for each bird, but to allow for any learning effects, perch type was not changed between the ten trials on each substrate.

From take-off force traces (S1 Fig), we extracted resultant velocities (m s^-1^), time (s) of force application, and angle of trajectory at toe-off (deg). Resultant velocities were calculated as in Provini et al. [12], by integrating the force with respect to time (from the time of the start of force application to toe-off). Time of force application was measured between the time after countermovement (when vertical force is less than body weight [12, 17]) and the time the bird’s feet were off the perch, confirmed visually in high-speed video. Angle of trajectory was calculated as the arctangent of the vertical force component divided by the horizontal force component. During landing, any measurements of timing and magnitude of instantaneous forces were made suspect by low frequency oscillations (see S2 Fig), but measuring timing to stability (s; identified as the final folding of the wings) was feasible.

To measure wing contributions to accelerations and decelerations in body velocity, a high-speed video (Photron 1024 PCI) was recorded laterally at 1,000 fps (shutter speed = 1/10,000). To test for velocity compensation by the wings following take-off, we measured flight velocity (m s^-1^) as the linear distance travelled by the body during the third wingbeat divided by the duration of that wingbeat. The difference between this flight velocity value and the resultant velocity at toe-off (measured from force traces) gave us the change in velocity during the first three wingbeats of flight. To test for potential variation in wing use among substrates in preparation for landing, we also measured the flight velocity during the final complete wingbeat before landing for three of the five birds. Angular velocities (rad s^-1^) for a representative individual were calculated by digitizing [18] two points on the bird: the eye and a point on the rump. The angle between horizontal and a line connecting these two points along the trunk axis of the bird was calculated. The angular velocity was then calculated as the change in that angle per second.

### Habitat use measurements

To measure perch selection versus availability in the wild, birds were observed at Philip Creek Cattle Station (Philip Creek, Northern Territory, Australia), and Burt Plain Cattle Station (Tanami Road, Alice Springs, Northern Territory, Australia), with permission from private landowners. Doves were located using visual and auditory cues. When a perched diamond dove was first encountered, the following information of the initial sighting was recorded: perch diameter and distance to fulcrum (determined as distance to an intersecting branch/trunk >5 cm in diameter, or the visually-identified main stalk of the plant). To compare bird sightings to available habitat [19], 7 photographs were taken of surrounding habitat, and diameters were measured using ImageJ for 200 randomly-selected points calibrated using a meter stick. Images were 4752x3168 pixels. To control for parallax effects upon perceived length in two-dimensional projects, we only selected branches for measurement that were in focus and within 5 m of the calibration stick. Points were randomized by dividing the image in to a 20x15 grid and using a random number generator in R version 3.3.2 [20] to identify the sampling grid within that grid. The first perch within the depth of field intersected from the top left corner of the grid square identified was measured.

### Data analysis

To test for differences in performance parameters between substrate type, we used a within-subjects, repeated-measures ANOVA, with individual bird as a factor. To compare available vs. used habitat type, we used a t-test. Analyses were performed in R [20]. We report means ± standard deviation.

## Results

### Take-off

**Fig 2:**
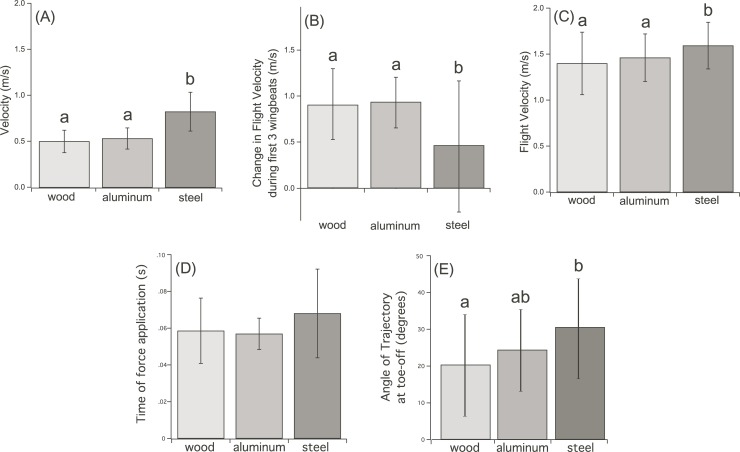
Take-off performance of diamond doves (*Geopelia cuneata*) on perches of various compliance. Wood was the most compliant, steel, the least. Letters indicate statistical significance of difference among means. (A) Resultant velocities at take-off due to the legs (B) Change in velocity during the first three wingbeats following take-off (C) Velocities at the third wingbeat of flight after toe-off. (D) Timing of force application during take-off (E) Angle of trajectory at take-off.

Take-off velocities were lower with more compliant perches. Take-off resultant velocities were significantly greater on the perch with the lowest compliance, steel (Fig 2A; ANOVA within-subjects factor F_2,8_ = 22.26, p<0.01). Resultant velocities produced during take-off from aluminum and wood perches were lower, and not statistically different from each other. Birds produced a velocity of 0.50 ± 0.12 ms^-1^ taking off from wood, followed by 0.53 ± 0.11 ms^-1^ from the aluminum perch, and 0.82 ± 0.21 ms^-1^ from the steel perch. The duration of time over which forces were applied did not change with substrate compliance, indicating that birds did not adjust timing of force application to improve take-off velocity in response to perch compliance. A general trend toward longer force application times was apparent, although not statistically significant (Fig 2D; F_2,8_ = 1.89, p = 0.21; wood = 0.06 ± 0.02 s, aluminum = 0.06 ± 0.01 s, steel = 0.07 ± 0.02). At take-off, the initial angle of the velocity vector differed between substrates, with more compliant substrates having lower initial take-off angles (Fig 2E; F_2,8_ = 5.45, p = 0.03; wood = 20.1 ± 13.7°, aluminum 24.2 ± 11.0°, steel = 30.3 ± 13.5°) Additionally, birds did not utilize perch recoil to recover potential energy from the perch as kinetic energy. In all instances, the bird lost contact with the perch before it recoiled (S1 Video). Likewise, the first downstroke was initiated immediately after losing contact with the perch, regardless of substrate. Birds may have compensated in flight for velocity lost on compliant substrates during take-off, but this trend was not significant in flight velocities. By the third wingbeat in flight, velocity not produced by the legs due to perch compliance remained statistically significant: doves taking off from a wood perch had an in-flight velocity of 1.40 ± 0.34 ms^-1^, take-off from an aluminum perch had a flight velocity at 1.46 ± 0.26 ms^-1^, and following steel at 1.59 ± 0.25 ms^-1^ (Fig 2C; F_2,8_ = 12.04, p<0.01). However, the change between take-off and in-flight velocities was significantly different between substrates (Fig 2B; F_2,8_ = 13.51, p = 0.003). Birds taking off from wood and aluminum perches recovered an average of 0.90 ± 0.39 ms^-1^ (wood) and 0.93 ± 0.27 ms^-1^ (aluminum) in the air while flights following take-off from steel produced 0.46 ± 0.71 ms^-1^ additional velocity by the third wingbeat.

### Landing

**Fig 3:**
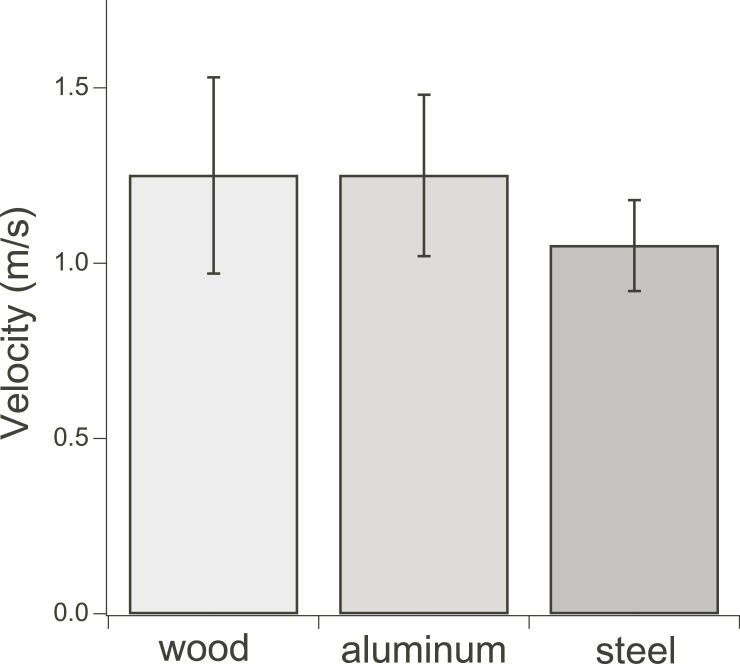
Flight velocities of diamond doves (*G*. *cuneata*) during the last complete wingbeat prior to landing for three perch types that vary in compliance.

During landing, birds approached perches at similar velocities, regardless of perch type, with statistically non-significant differences in mean velocity (F_2,4_ = 1.36, p = 0.35) (Fig 3). Thus, they did not prepare for landing to capitalize on the physical properties of known substrates. In contrast, stabilization and control were greatly challenged on compliant substrates during landing.

**Fig 4:**
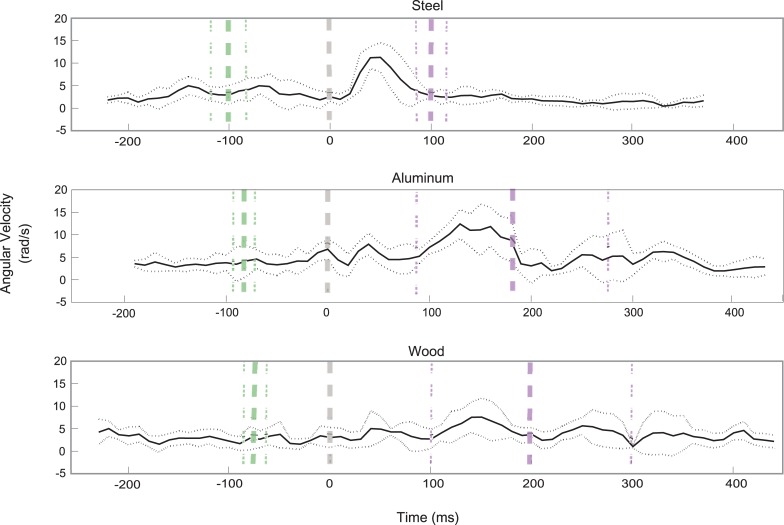
Average angular velocities of a representative individual (Dove 46) during landing on different substrates (N = 10 trials per substrate). Average timing (solid line) +/- standard deviation (dashed lines) is noted for foot extension (green), landing (gray), and the point the bird is considered stable (purple), following body reorientation. Instabilities are notable in following landing on the compliant substrates (aluminum and wood), indicated by additional angular velocities.

Upon landing, birds produced predictable patterns of body reorientation. At touch-down, the bird immediately adjusted to a more upright posture, and then folded the wings–as observed upon landing on the substrate with minimal-compliance, steel (Fig 4). During this reorientation phase, the bird initially touched down at a body angle between the eye and rump of 26.3 ± 45.8° from horizontal. The bird then reoriented to a resting angle of 70.4±2.8° from horizontal. During the reorientation phase, the bird’s peak angular velocity of reorientation occurred at 50 milliseconds after touch-down, at 11.4±3.3 rad s^-1^.

A failure in stabilizing, then, was defined as when this reorientation was delayed or variable. In both wood and aluminum treatments, birds took considerably longer to reach stability, and showed high standard deviations in the timing of stability (Fig 4B and 4C). Further, additional changes in angular velocity before and after stability indicate failed attempts or drastic posture changes in order to gain stability. During landings on wood, we found many small angular velocity peaks–the first 4.8±4.0 rad s^-1^ at approximately 50 milliseconds following touchdown, then 7.4 ± 4.2 rad s^-1^ at 150 milliseconds, and again 5.4±1.9 rad s^-1^ at 250 milliseconds, and finally at 4.3±2.1 rad s^-1^ at 410 milliseconds. During the final three angular velocity peaks, body angle relative to horizontal varied between 52.9° and 83.0°. During landings on aluminum, small peaks in angular velocity were apparent as well, at 7.6± 2.4 rad s^-1^ at 40 milliseconds after touchdown, again at 12.1±3.2 rad s^-1^ at 130 milliseconds, and 6.0±2.3 rad s^-1^ at 330 milliseconds. During the final two angular velocity peaks, the body angle varied between 63° and 84°.

**Fig 5:**
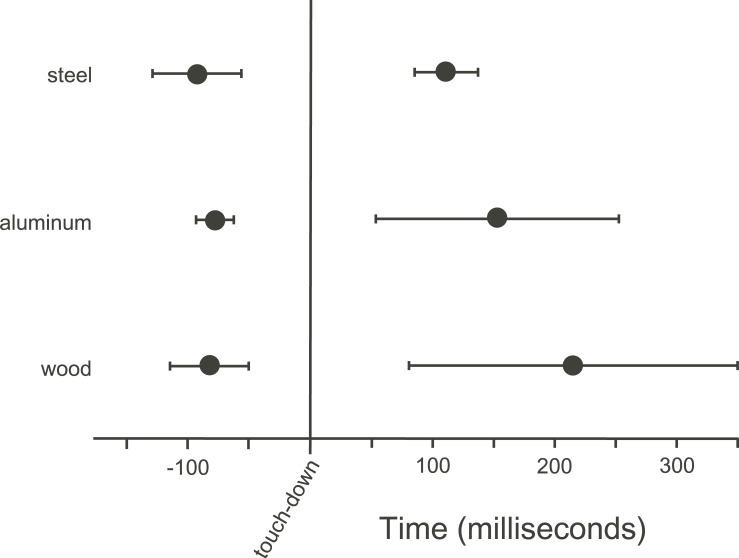
Timing of leg extension prior to landing (left) and point at which diamond doves (*G*. *cuneata*) reoriented and were stable (right), relative to touchdown (time zero). Instabilities during landing were notable due to a longer time following touchdown and higher standard deviation until stabilization.

There was a difference between time-to-stabilization between substrate treatments (Fig 5; F_2,8_ = 4.21, p = 0.056). On wood substrates, birds were stable at 213 ± 134 ms following touch-down, on aluminum, 151 ± 99 ms, while stabilization on steel occurred at 109 ± 26 ms following touch-down. The large standard deviations among substrate treatment indicate challenges of gaining control. In contrast, we found no significant differences among perch types for timing of leg extension prior to landing, providing additional evidence that the birds did not effectively modulate landing preparation (Fig 5; F_2,8_ = 0.59, p = 0.58).

**Fig 6:**
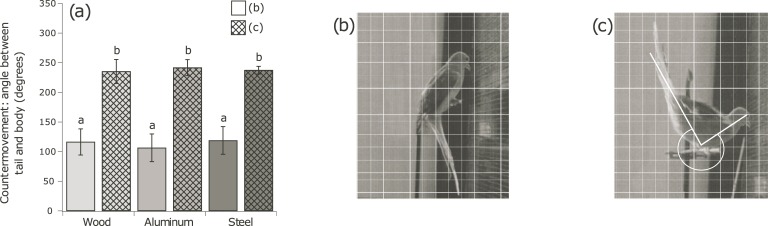
(A) Angle of countermovements during landing to aid in stabilization for both countermovement conditions on all substrate types. (B) An example of a tail-under countermovement, reaching an average angle of 114 ± 24 degrees. (C) An example of a tail-over countermovement, reaching an average angle of 237 ± 17 degrees. Effects of perch compliance were not statistically significant between substrates for both tail-under and tail-over countermovements (P > 0.17).

Stereotypical countermovements following landing contributed to regaining stability. Birds generally (74%) used their tail as a ventral counter weight (Fig 6A & 6B), flexing the tail ventrally under the perch to help counter the rotational motion of the body. In 26% of the trials, the birds used their tails in the opposite fashion, extending them dorsally over their heads and vertically above the perch (Fig 6A and 6C). This counter-weight function was observed most frequently on the wood perch (50% of the time), the most compliant substrate (S2 Video), followed by 17% of the time when landing on aluminum, and 10% of the time when landing on steel. Within each class of countermovement, the angle between the bird’s body and tail did not differ significantly among substrates (tail-down: 0.17<p<0.94, tail-up: 0.78<p<0.98; Fig 6A).

### Perch preference in free living birds

**Fig 7:**
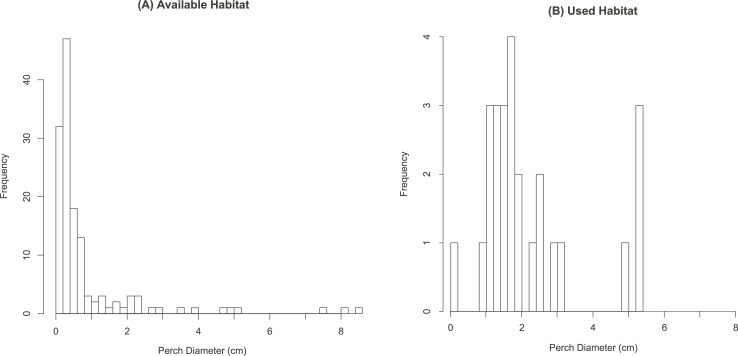
Perch selection in wild diamond doves in Australia (*G*. *cuneata*; *N* = 25). (A) Perch diameters available within the habitat (B) Diameters of perches where doves were observed.

There were significant differences between perch diameters available in the habitat and the perch diameter upon which diamond doves were found (t-test p <0.01; Fig 7). Birds were located on perches averaging 2.3 ± 1.4 cm in diameter, with a median value of 1.7 cm (n = 25; Fig 7A). The smallest perch found in-use was 0.9 cm in diameter, and the largest was 5.4 cm. In contrast, the average perch available in the habitat was 0.9 ± 1.4 cm in diameter. The minimum diameter observed was 0.1 cm in diameter, while the maximum was 8.5 cm. Birds were observed on average 73.8 ± 55.1 cm from the nearest fulcrum within a shrub or tree.

## Discussion

Increased perch compliance adversely affects both take-off and landing abilities. Contrary to our main hypothesis that the apparent mastery of take-off and landing would be related to a capacity to harvest elastic energy and modulate kinematics, diamond doves did not capitalize on perch compliance during transitions. Transitions on compliant substrates featured lower velocity during take-off, and no change in approach kinematics, alongside long periods of restabilization during landing. Instead of coordinated use of the hindlimb and forelimb modules, the doves used these modules independently, such that lower initial velocity due to the legs impacted later flight velocities, and during landing the wing and tail modules were used to aid in stabilization. Given the problems posed by compliance, it is understandable that free-living birds may preferentially select perches of larger diameter, thereby minimizing effects of compliance during locomotor transitions.

### Compliance affects velocity during take-off

During take-off, birds did not recover from lower starting velocities following take-off from perches with higher compliance. We found no evidence that birds modulate leg function to adjust for perch compliance; birds did not adjust the timing of force production (Fig 2B) or take-off force, as suggested by [14]. Thus, perches with higher compliance showed a lower instantaneous velocity at toe-off. Mathematical models of humans, bush-babies, and insects suggest that altering kinematic strategies on substrates of various compliance can dramatically change the resulting jump height [5]. However, we saw no evidence for altering force-application timing during take-off in the doves. Both anole lizards [8] and click-beetles [21] also do not alter force application to account for substrate variation.

### Compliance affects timing and stability during landing

During landings, we observed changes in body orientation consistent with instabilities on compliant substrates (Fig 4), and subsequent time to stability was longer on the more compliant (wood, aluminum) substrates (Fig 5). Birds were often observed using either or both their tail (Fig 6) and wings to aid in restablization. Extending limbs to improve stability is well established in humans, capitalizing on displacing the center of mass to align with the ground reaction force [22]. Tails in vertebrates contribute to stability during terrestrial and aerial locomotion [23]. Wing flapping also displaces the center of mass, but it can capitalize on aerodynamic force production as well [24]. The balance between aerodynamic and inertial torque during stabilization from a kinematic, neuro-mechanic, and sensory perspective is a rich area for future studies.

While these instabilities delayed time taken to land, birds were stable in less than 0.3 seconds. One method to stabilize more quickly would be to rely on the wings for greater decelerations prior to landing, much like the hummingbird [15]. This would minimize the ground reaction force, and orient the remaining force in line with the center of mass on impact. Such a landing would place greater energetic costs on the bird, as it would increase time spent in slow near-hovering flight—the energetically most expensive flight speed [10]. By bypassing the slowest flight speeds and allowing the legs and perch to decelerate the remaining speed, the energetic costs of hovering flight are avoided. Countermovements of the wing and tail may incur some costs, but are much slower than the costs of flight. Further, the fitness implications of slight delays to stability may be null, as birds were all capable of successful landings.

### Wing & leg modules function independently

Wing and leg modules [11] were found to function largely independently during take-off, consistent with other studies of take-off in birds other than hummingbirds [12, 13, 15, 17]. During take-off, the legs transfer energy to the perch that is not regained, causing initial low velocities. Following take-off, the wings increase flight velocity differentially, with lower initial take-off velocities having higher compensatory flight velocities during the first three wingbeats (Fig 2B). However, flight velocity by the third wingbeat remains inhibited by the initial lower velocities on compliant substrates (Fig 2C). During landing, the wings decelerate birds to a consistent flight velocity before touchdown.

However, following touchdown, both the wings and the tail (a third module) assist the legs in counterbalancing the body to improve stability. This novel tri-partite recruitment of modules is distinct from the walking and flying patterns reported in Gatesy and Dial [11] where the tail is linked to the wings during flight and to the legs during walking. Neurological or musculo-skeletal limitations offer a hypothesis to explain the staggering of module timing, as well as the inability to compensate for perch compliance. Transitions may be constrained by a neurological central pattern generator [25, 26], wherein the timing between leg- and wing-modules cannot be adjusted. Further work exploring the neurobiology behind modules is necessary to improve our understanding of potential neuromechanical limitations.

For birds, maximum velocity produced by the legs during take-off may not be crucial, as the wings take over immediately following loss of contact with the ground [12]. The dominant flight muscle, the pectoralis, produces extremely large mass-specific power output [10, 27]. Recent studies have established that the aerodynamic forces produced during energetically demanding slow flight [10] are modulated [12, 13, 27] such that even during the most energetically demanding phase, power output can be adjusted depending on acceleration requirements. As such, it is arguable that the avian wing musculo-skeletal system is relatively ‘overbuilt’ in comparison to the leg, and so, is more capable of adjusting and compensating during transitions than the legs. While this is not observed in our experiments, it remains feasible for flights of longer duration than the short-distances we explored in the present study.

### Free-living birds prefer non-compliant perches

Doves preferentially selected larger-diameter perches than in our laboratory tests (Fig 7). This may aid in compensating for inefficient highly compliant substrates (Figs 2 and 4). Our field measurements were unable to include a direct measure of compliance of the substrates, and our analysis relies on the significant assumption that diameter is representative of compliance on a rough scale. Given that compliance varies greatly between plant species [1], our use of diameter is an oversimplification. Further comparative work is warranted to examine the generalities of perch use in birds, alongside detailed habitat characteristic quantification (such as [9]).

Birds used in the laboratory study were raised in indoor aviaries with unlimited food supply and no predation pressure. They were not necessarily motivated to maximize performance in take-off or landing, so caution is warranted when applying the results toward an understanding of transitions between substrates and the air in the wild. While the transitions we measured here did not optimize for variation in perch compliance, all birds completed their transitions without injury. During take-off, birds were able to partially compensate in flight. During landing, all birds decelerated and reached stability successfully. Though the transitions were not efficient in terms of energy use and velocity, they were effective in accomplishing the desired task. Further comparative studies are necessary to understand if inefficient transitions are a general characteristic of birds, compensated using powerful flight muscles.

## Supporting information

S1 Fig Traces of instantaneous forces as recorded by the force perch for representative trial take-offs on (A) wood, (B), aluminum, and (C), steel. Solid black is vertical force (F_V_), dashed blue is horizontal force (F_H_), red vertical line represents time at which the bird loses contact with the perch.
(EPS)

S2 Fig Traces of instantaneous forces as recorded by the force perch for representative trial landings on (A) wood, (B), aluminum, and (C), steel. Solid black is vertical force (F_V_), dashed blue is horizontal force (F_H_), red vertical line represents time at which contact with the force perch is initiated.
(EPS)

S1 Video Representative trial for take-off from the perch with the highest compliance (wood). High-speed video was recorded at 1000 Hz.
(MP4)

S2 Video Representative trial for landing on the perch with the highest compliance (wood). High-speed video was recorded at 1000 Hz.
(MP4)

S1 File All data associated with this manuscript. (XLSX)
